# Exosomes secreted by palmitic acid-treated hepatocytes promote LX-2 cell activation by transferring miRNA-107

**DOI:** 10.1038/s41420-021-00536-7

**Published:** 2021-07-07

**Authors:** Wei Wang, Fangfang Li, Xiaoyang Lai, Han Liu, Shuting Wu, Yunqin Han, Yunfeng Shen

**Affiliations:** Department of Endocrinology and Metabolism, Institute for the Study of Endocrinology and Metabolism in Jiangxi Province, The Second Affiliated Hospital of Nanchang University, 330006 Nanchang, China

**Keywords:** Molecular biology, Diseases

## Abstract

Activation of hepatic stellate cells (HSCs) is a key inducer of liver fibrogenesis in nonalcoholic fatty liver disease (NAFLD). Exosomes play an important role between hepatocytes and HSCs. This study aims to explore the role of exosomes derived from palmitic acid (PA)-treated hepatocytes in regulating HSCs (LX-2 cell) proliferation and activation and the underlying mechanisms. Exosomes were isolated from PA-treated human normal hepatocytes and incubated with LX-2 cells. Cell Counting Kit-8 (CCK-8) was performed to determine LX-2 cell proliferation, and the expression of fibrosis markers α-smooth muscle actin (α-SMA) and collagen type 1 α1 (CoL1A1) were examined to evaluateLX-2 cell activation. PA induced hepatocytes to release more exosomes enriched in miR-107. Mechanically, on the one hand, exosomes from PA-treated hepatocytes shuttled miR-107 to LX-2 cells, where miR-107 activated Wnt signaling by targeting DKK1 and thereby induced LX-2 cell activation; on the other hand, PA-treated hepatocytes derived exosomes also delivered miR-107 to CD4 + T lymphocytes, where miR-107 elevated IL-9 expression by targeting Foxp1, which bound to the IL-9 promoter in CD4 + T cells and suppressed Th9 cell differentiation and reduced IL-9 expression, and thus promoted LX-2 cell activation by activating Raf/MEK/ERK signaling pathway.

## Introduction

Non-alcoholic fatty liver disease (NAFLD) is a known inducer of chronic liver disease and has became a common public health problem. NAFLD begins with steatosis and advances to nonalcoholic steatohepatitis (NASH), fibrosis, and ultimately cirrhosis [1]. As part of NAFLD spectrum, liver fibrosis is a common scarring response to various forms of chronic liver injury. Hepatic stellate cells (HSCs) activation (the transdifferentiation of vitamin-A-storing cells into fibrogenic myofibroblasts) is a dominating driver of liver fibrogenesis [2, 3]. Thus, understanding the mechanisms underlying HSCs activation is of great significance for therapeutic intervention.

Exosomes are small (30 to 100 nm) extracellular vesicles that are implicated in various diseases. Exosomes can deliver important biological molecules (such as lipids, proteins, and RNAs) to recipient cells, thus mediating intercellular signal transduction [4]. Various liver resident cells including hepatocytes, HSCs, and macrophages, can secrete exosomes, or act as target cells of exosomes [5–7]. Liver fibrosis involves the interaction between HSCs, hepatocytes, and immune cells. Exosomes serve as carriers for intercellular communication between these cell populations, which play an important role in regulating HSCs phenotypic switch and liver fibrosis [8].

Among typical exosomal cargos, microRNAs (miRNA) have a critical role in regulating posttranscriptional gene expression [9]. A microarray analysis of differentially expressed miRNAs revealed that miR-107 expression was obviously upregulated in PA-treated hepatocytes derived exosomes [10]. In addition, miR-107 was highly expressed in the livers of the Stelic Animal Model (STAM) mice at steatotic, NASH-fibrotic, and hepatocarcinoma stages of liver carcinogenesis [11]. Moreover, exosomes derived from PA-treated hepatocytes upregulated fibrotic genes expression in HSCs [10]. Based on this evidence, we hypothesized that exosomes secreted by PA-treated hepatocytes may modulate HSC phenotype by miR-107 delivery.

In this study, our results demonstrated that exosomes derived from PA-treated hepatocytes facilitated LX-2 cell proliferation and activation via transferring miR-107, and the underlying mechanisms were also determined.

## Results

### Characterization of hepatocytes-derived exosomes

**Fig. 1:**
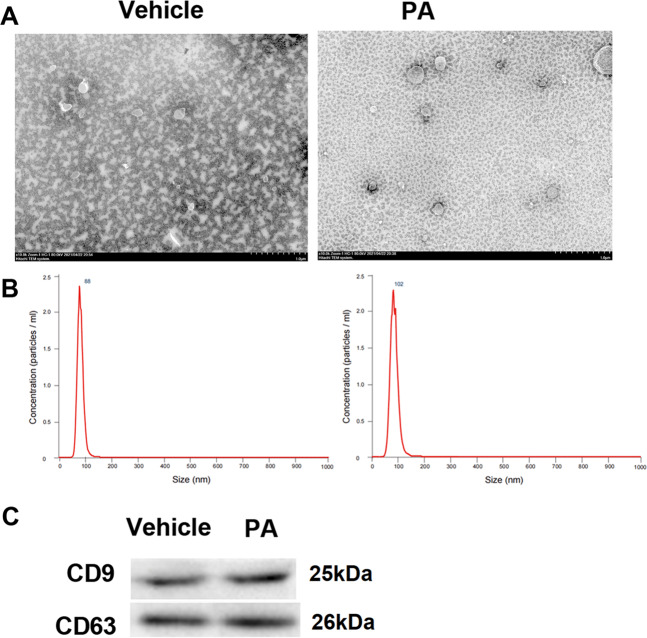
Characterization and identification of exosomes from PA-treated hepatocytes. Exosomes were isolated from culture media of vehicle-treated or palmitic acid (PA; 0.4 mM; 16 h)-treated hepatocytes. **A** TEM was used to observe the morphology of exosomes (Scale bar = 100 nm). **B** Nanoparticle tracking analysis was performed to analyze the exosomes. **C** Western blot was performed to detect exosomal surface markers CD9 and CD63.

The exosomes isolated from hepatocytes were characterized by TEM, nanoparticle tracking analysis, and western blot. TEM revealed that the exosomes in both groups were round-shaped (Fig. 1A). Data from nanoparticle tracking analysis further confirmed the exosomes isolated from different hepatocytes, and the results suggested that PA induced more exosomes (102 particles/ml), compared with the control (88 particles/ml) (Fig. 1B). Moreover, Western blot results showed that exosomes from vehicle-and PA-treated hepatocytes were both positive for the exosomal surface markers (CD9, CD63) (Fig. 1C). Fortunately, CD9 and CD63 were undetected in the cell media treated with PA (Supplementary 1).

### Exosomes from PA-treated hepatocytes induces LX-2 cell activation by transferring miR-107 to LX-2 cells

**Fig. 2:**
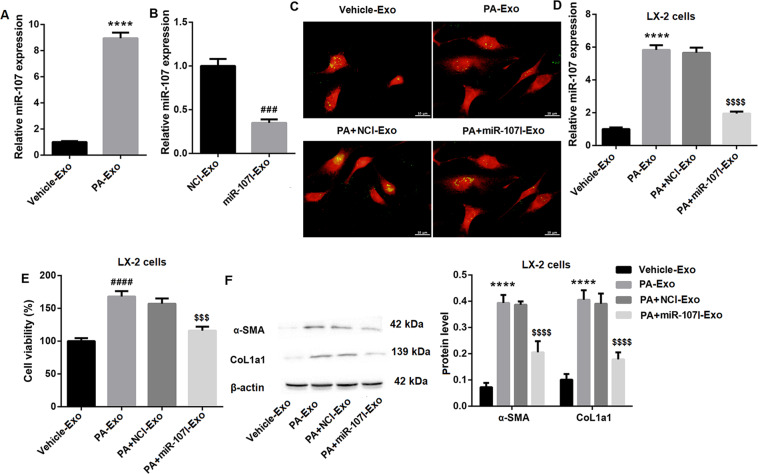
PA-Exo induces LX-2 cell activation by transferring miR-107 to LX-2 cells. **A** QRT-PCR was performed to detect miR-107 expression in Vehicle-Exo and PA-Exo. **B** QRT-PCR was performed to determine the transfection efficiency of miR-107I in hepatocytes. The hepatocytes-derived exosomes were divided into Vehicle-Exo, PA-Exo, PA + NCI-Exo, and PA + miR-107I-Exo, then LX-2 cells were cocultured with them for 16 h, respectively. **C** The hepatocytes were labeled with the lipophilic fluorescent dye DiO (green) and co-incubated with LX-2 cells transfected with mCherry plasmid (red). The laser confocal microscope was used to analyze the intake of exosomes by LX-2 cells (Scale bar = 10 μm). QRT-PCR, CCK-8, and western blot were performed to detect miR-107 expression (**D**), cell viability (**E**), and the protein levels of α-SMA and CoL1a1 (**F**) in LX-2 cells co-incubated with Vehicle-Exo, PA-Exo, PA + NCI-Exo, and PA + miR-107I-Exo, respectively. ^****^P < 0.0001 vs. Vehicle-Exo; ^###^*P* < 0.001 vs. miR-107I-Exo; ^$$$^*P* < 0.001 vs. PA + NCI-Exo; ^$$$$^*P* < 0.0001 vs. PA + NCI-Exo. Data were expressed as the mean ± standard deviation (*n* = 3).

Our results also revealed that, compared to Vehicle-Exo, miR-107 expression was significantly upregulated in PA-Exo (Fig. 2A). To determine whether PA-Exo can regulate cell behavior of recipient HSCs (LX-2) via transferring miR-107, LX-2 cells were cocultured with Vehicle-Exo, PA-Exo, PA + NCI-Exo, and PA + miR-107I-Exo. Firstly, qRT-PCR results showed that miR-107I notably knocked down miR-107 expression (Fig. 2B), indicating that miR-107 was effectively deleted. Also, the uptake of hepatocytes-derived exosomes by LX-2 cells was determined by a laser confocal microscope (Fig. 2C). Moreover, miR-107 expression in LX-2 cells was greatly increased after PA-Exo treatment but was significantly decreased following PA + miR-107I-Exo treatment (Fig. 2D). These data together suggested that PA induced the transfer of miR-107 from hepatocytes to LX-2 cells via exosomes.

Next, we sought to determine whether PA-Exo regulates LX-2 cell activation by transferring miR-107. The results showed that PA-Exo treatment significantly facilitated LX-2 cell activation, as evidenced by enhanced cell proliferation (Fig. 2E) and elevated protein levels of α-smooth muscle actin (α-SMA) and type I collagen (CoL1a1) (Fig. 2F**)** in LX-2 cells. While, transfection with miR-107I in hepatocytes effectively abrogated PA-Exo-induced LX-2 cell activation (Fig. 2E, F**)**. These data indicated that PA-Exo induced LX-2 cell activation by transferring miR-107.

### MiR-107 induces LX-2 cell activation through inhibiting DKK1 expression and activating Wnt signaling

**Fig. 3:**
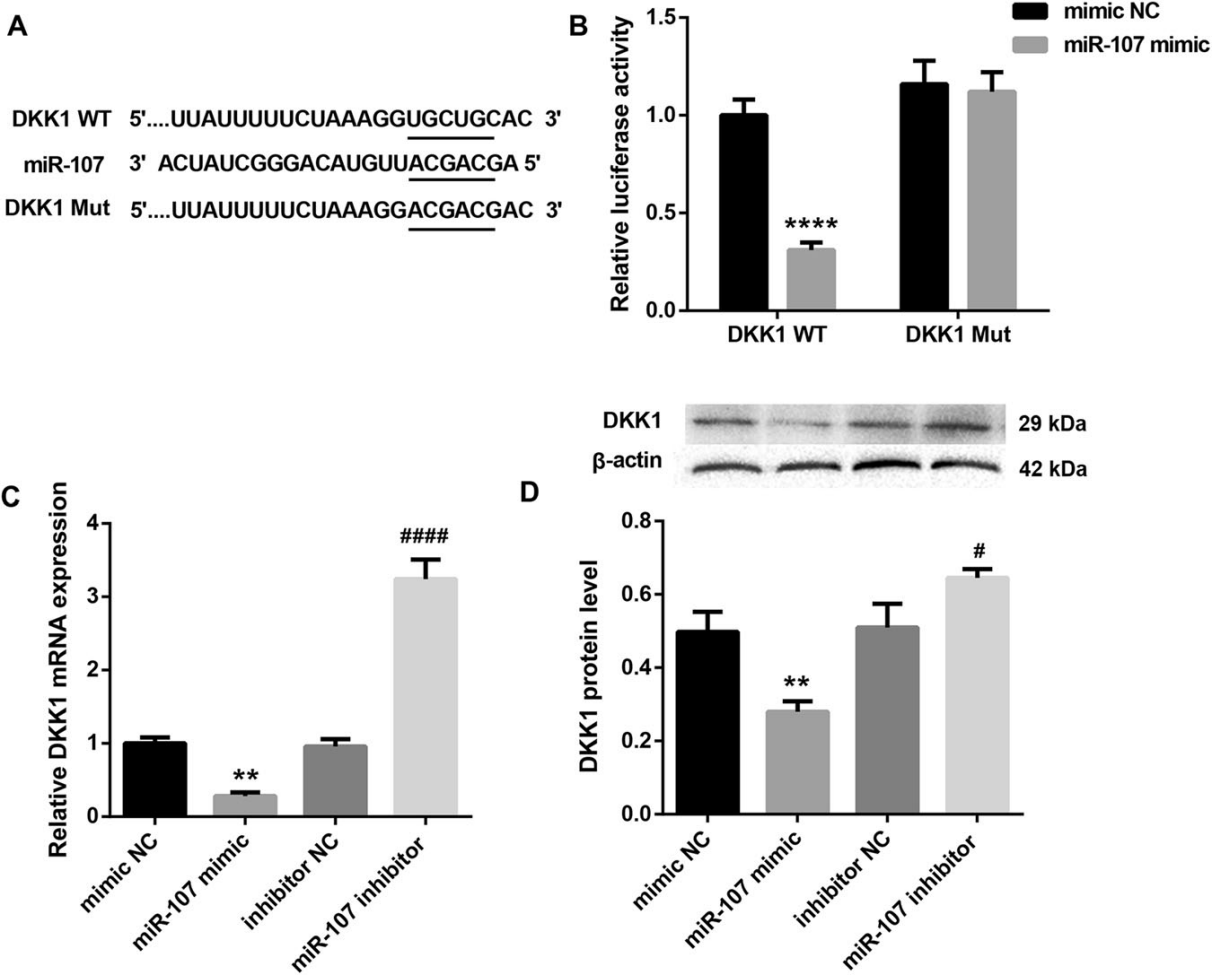
MiR-107 targets DKK1. **A** The bioinformatics analysis of the binding sites between miR-107 and DKK1. **B** Luciferase report assay was performed to analyze the binding of DKK1 and miR-107. QRT-PCR and western blot were performed to examine the mRNA (**C**) and protein (**D**) levels of DDK1 in LX-2 cells, respectively. ^**^P < 0.01, ^****^P < 0.0001 vs. mimic NC; ^#^*P* < 0.05, ^####^*P* < 0.0001 vs. inhibitor NC. Data were expressed as the mean ± standard deviation (*n* = 3).

**Fig. 4:**
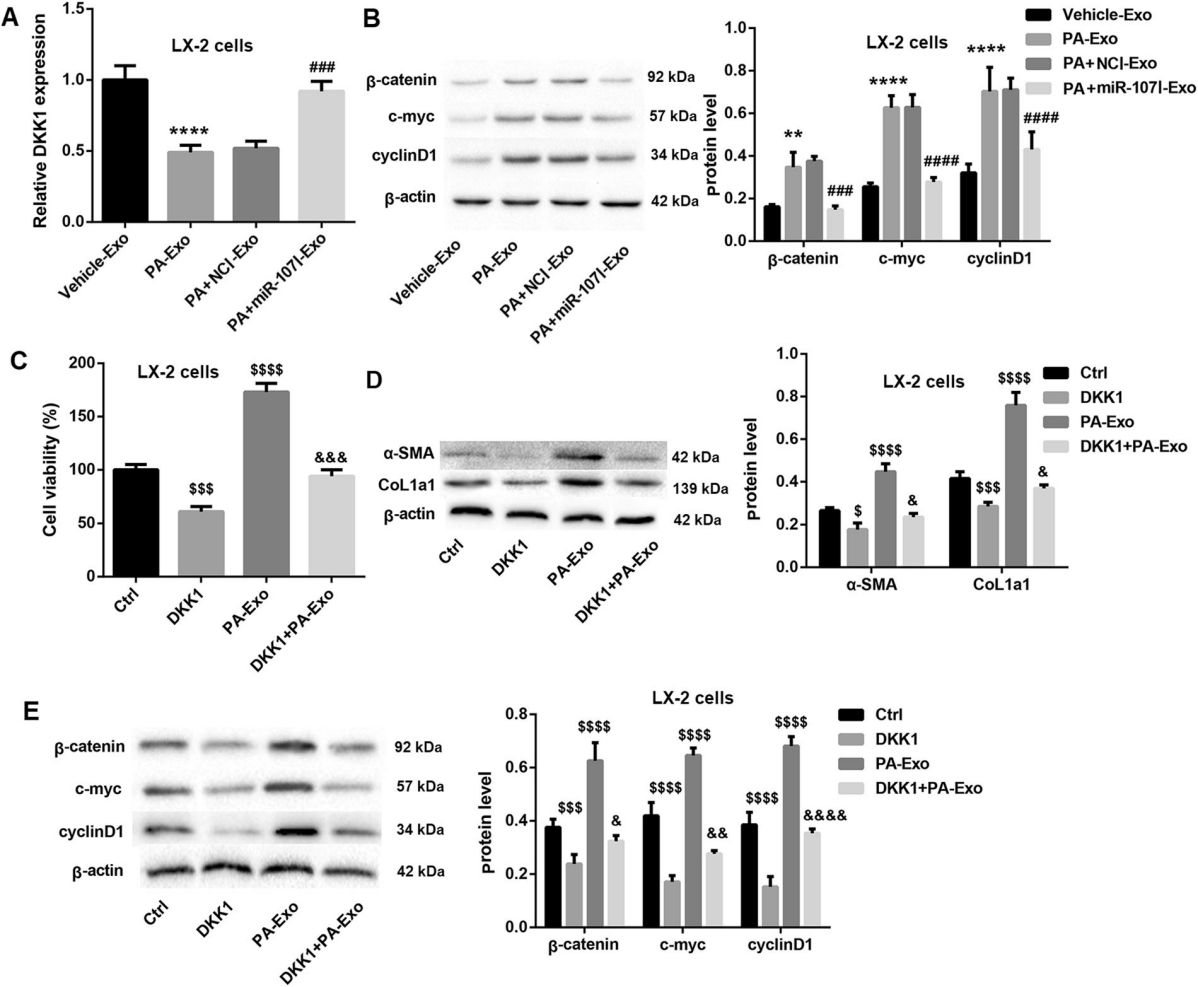
MiR-107 induces LX-2 cell activation through inhibiting DKK1 expression and activating Wnt signaling in LX-2 cells. LX-2 cells were cocultured with hepatocytes-derived exosomes (Vehicle-Exo, PA-Exo, PA + NCI-Exo, and PA + miR-107I-Exo) for 16 h, respectivily. QRT-PCR and western blot were performed to examine the mRNA expression of DKK1 (**A**) and protein levels of β-catenin, c-myc, and cyclinD1 (**B**) in LX-2 cells. LX-2 cells were transfected with pcDNA3.1-DKK1 or empty vector, and followed by PA-Exo treatment. CCK-8, and western blot was performed to evaluate LX-2 cell viability (**C**), the protein levels of α-SMA and CoL1A1 (**D**), and the protein levels of β-catenin, c-myc, and cyclinD1 (**E**) in LX-2 cells. ^**^P < 0.01, ^****^P < 0.0001 vs. Vehicle-Exo; ^###^*P* < 0.001, ^####^*P* < 0.0001 vs. PA + NCI-Exo; ^$^*P* < 0.05, ^$$$^*P* < 0.001, ^$$$$^*P* < 0.0001 vs. Ctrl; ^&^*P* < 0.05, ^&&^*P* < 0.01, ^&&&^*P* < 0.001, ^&&&&^*P* < 0.0001 vs. DKK1. Data were expressed as the mean ± standard deviation (*n* = 3).

Next, we explored the potential mechanism by which miR-107 induced LX-2 cell activation. DKK1 is an antagonist of Wnt pathway [12] that promotes liver fibrosis by enhancing HSCs activation [13]. Targetscan suggested that DKK1 was a putative target of miR-107 (Fig. 3A). Thus, we speculated whether miR-107 induced HSCs activation via inhibiting DKK1 and activating Wnt signaling pathway. Luciferase reporter assay showed that, compared with mimic NC, miR-107 mimic markedly decreased the luciferase activity in DKK1 WT reporter, whereas had no effect on DKK1 Mut reporter (Fig. 3B), suggesting that DKK1 is directly targeted by miR-107. Furthermore, miR-107 mimic significantly increased DKK1 mRNA and protein levels, whereas miR-107 inhibitor yielded the opposite results (Fig. 3C, D). In addition, DKK1 mRNA expression in LX-2 cells was notably inhibited after PA-Exo treatment, but was significantly elevated in PA + miR-107I-Exo group (Fig. 4A). Moreover, PA + miR-107I-Exo efficiently decreased the PA-Exo-induced protein levels of β-catenin, c-myc, and cyclinD1 (Wnt signaling-related proteins) in LX-2 cells (Fig. 4B). Above results suggested that PA-Exo might inhibit DKK1 expression and activate Wnt pathway in LX-2 cells by transferring miR-107 to LX-2 cells.

To further determine whether DKK1 was involved in PA-Exo-mediated activation of Wnt signaling and HSCs activation in LX-2 cells, LX-2 cells were transfected with pcDNA3.1-DKK1 or empty vector, followed by PA-Exo treatment. Data revealed that DDK1 overexpression in LX-2 cells caused a notable decrease in cell proliferation (Fig. 4C) and protein levels of α-SMA and CoL1a1 (Fig. 4D), indicating that DKK1 overexpression suppresses LX-2 cell activation. Furthermore, DDK1 overexpression significantly abolished the PA-Exo-mediated promotion of cell proliferation (Fig. 4C) and elevated protein levels of α-SMA and CoL1a1 (Fig. 4D) in LX-2 cells. In addition, DKK1 overexpression notably reduced the PA-Exo-mediated induction of protein levels of β-catenin, c-myc, and cyclinD1 in LX-2 cells (Fig. 4E). Collectively, these data suggested that PA-Exo activated Wnt signaling and LX-2 cell activation via inhibiting DKK1 expression in LX-2 cells.

### Exosomes from PA-treated hepatocytes induces Th9 cell differentiation and IL-9 expression by transferring miR-107 to CD4^+^ T cells

**Fig. 5:**
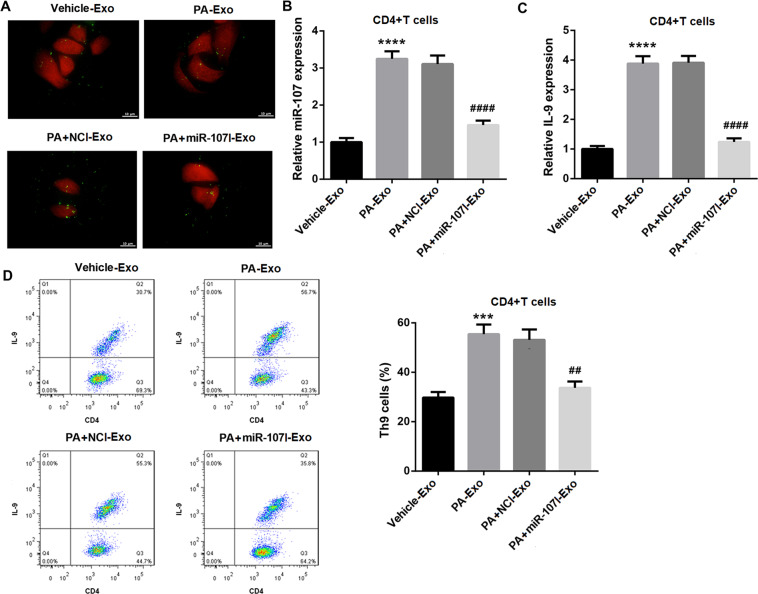
PA-Exo induces Th9 cell differentiation and IL-9 expression by transferring miR-107 to CD4^**+**^ T cells. Naive CD4^+^ T cells were treated with 10 ng/mL human IL-4, 1 ng/mL human TGF-β, and 10 μg/mL anti-IFN-γ monoclonal antibodies to induce Th9 differentiation, and followed by the coculture with hepatocytes-derived exosomes (Vehicle-Exo, PA-Exo, PA + NCI-Exo, and PA + miR-107I-Exo). **A** The hepatocytes were labeled with the lipophilic fluorescent dye DiO (green) and co-incubated with CD4^+^ T cells transfected with mCherry plasmid (red). The laser confocal microscope was used to analyze the intake of exosomes by CD4^+^ T cells (Scale bar = 10 μm). QRT-PCR and flow cytometry were performed to detect the mRNA expression of miR-107 (**B**) and IL-9 (**C**), and Th9 cells proportion (**D**) in CD4^+^ T cells. ^***^P < 0.001, ^****^P < 0.0001 vs. Vehicle-Exo; ^##^*P* < 0.01, ^####^*P* < 0.0001 vs. PA + NCI-Exo. Data were expressed as the mean ± standard deviation (*n* = 3).

HSCs activation can be regulated by immune system [14, 15]. Recent evidence indicates that Th9 cells and its signature cytokine IL-9 play a deleterious role in increasing hepatic fibrosis [16]. Thus, we guessed whether PA-Exo regulated Th9 cell differentiation via transferring miR-107. The laser confocal microscope confirmed the uptake of hepatocytes-derived exosomes by CD4^+^ T cells (Fig. 5A). MiR-107 expression in CD4^+^ T cells was significantly increased after PA-Exo treatment, but was significantly decreased in PA + miR-107I-Exo group (Fig. 5B), suggesting that PA induced the transfer of miR-107 from hepatocytes to recipient CD4^+^ T cells via exosomes. Furthermore, PA-Exo treatment markedly induced IL-9 mRNA expression (Fig. 5C) and Th9 cells proportion (Fig. 5D) in CD4^+^ T cells. Importantly, the effect of PA-Exo was effectively abrogated after the transfection with miR-107I in hepatocytes (Fig. 5C, D). These data together suggested that PA-Exo induced Th9 cell differentiation and IL-9 expression by transferring miR-107 to CD4^+^ T cells.

### MiR-107 induces Th9 differentiation and IL-9 expression via inhibiting Foxp1 in CD4^+^ T cells

**Fig. 6:**
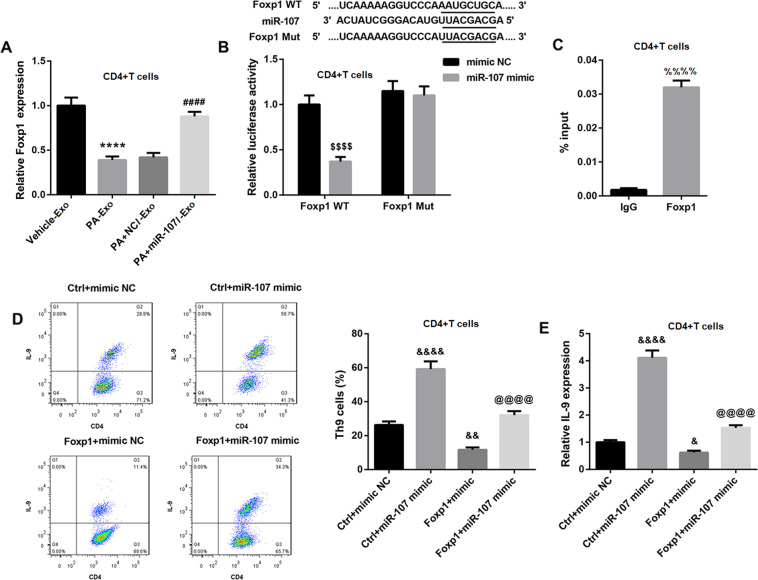
MiR-107 induces Th9 differentiation and IL-9 expression via inhibiting Foxp1 in CD4^**+**^ T cells. **A** Naive CD4^+^ T cells were treated with 10 ng/mL human IL-4, 1 ng/mL human TGF-β, and 10 μg/mL anti-IFN-γ monoclonal antibodies to induce Th9 differentiation, and followed by the coculture with hepatocytes-derived exosomes (Vehicle-Exo, PA-Exo, PA + NCI-Exo, and PA + miR-107I-Exo). QRT-PCR was performed to examine the mRNA expression of Foxp1 in CD4^+^ T cells. **B** Luciferase report assay was performed to determine the binding of Foxp1 and miR-107. **C** CHIP was carried out to analyze the binding of Foxp1 and IL-9 promoter in CD4^+^ T cells. Flow cytometry and qRT-PCR were performed to examine Th9 cells proportion (**D**), and IL-9 mRNA expression (**E**) in CD4^+^ T cells. ^****^P < 0.0001 vs. Vehicle-Exo; ^####^*P* < 0.0001 vs. PA + NCI-Exo; ^$$$$^*P* < 0.0001 vs. mimic NC; ^%%%%^*P* < 0.0001 vs. IgG; ^&^*P* < 0.05, ^&&^*P* < 0.01, ^&&&&^*P* < 0.0001 vs. Ctrl+ mimic NC; ^@@@@^*P* < 0.0001 vs. Ctrl+ miR-107 mimic. Data were expressed as the mean ± standard deviation (*n* = 3).

We then examined the mechanism by which miR-107 induced Th9 differentiation and IL-9 expression in CD4^+^ T cells. Evidence indicates that Foxp1 binds to the IL-9 promoter in naive CD4^+^ T cells and inhibits IL-9 expression, and negatively regulates Th9 cell differentiation [17]. Targetscan further revealed that Foxp1 was a putative target of miR-107. Thus, we sought to elucidate whether miR-107 induced Th9 differentiation and IL-9 expression via inhibiting Foxp1. To address this, we examined Foxp1 expression in CD4^+^ T cells following PA-Exo treatment. Foxp1 mRNA expression was significantly decreased in CD4^+^ T cells after PA-Exo treatment, but was restored in PA + miR-107I-Exo group (Fig. 6A). Furthermore, luciferase reporter assay showed a marked decrease in luciferase activity in Foxp1 WT reporter after the introduction of miR-107 mimic when compared with mimic NC (Fig. 6B), verifying that Foxp1 was directly targeted by miR-107. These data indicated that PA-Exo inhibited Foxp1 expression in CD4^+^ T cells by transferring miR-107.

Furthermore, CHIP assay displayed that IL-9 promoter was enriched with Foxp1 in CD4^+^ T cells (Fig. 6C), confirming the binding of Foxp1 to the IL-9 promoter. In addition, in CD4^+^ T cells, Foxp1 overexpression led to a notable decrease in Th9 proportion (Fig. 6D) and IL-9 mRNA expression (Fig. 6E). Notably, Foxp1 overexpression effectively abolished the miR-107 mimic-mediated increase in Th9 proportion (Fig. 6D) and IL-9 mRNA expression (Fig. 6E). Taken together, these findings indicated that miR-107 induced Th9 differentiation and IL-9 expression via inhibiting Foxp1 in CD4^+^ T cells.

### Exosomes from PA-treated hepatocytes induces LX-2 cell activation through miR-107-mediated upregulation of IL-9 expression in CD4^+^ T cells

**Fig. 7:**
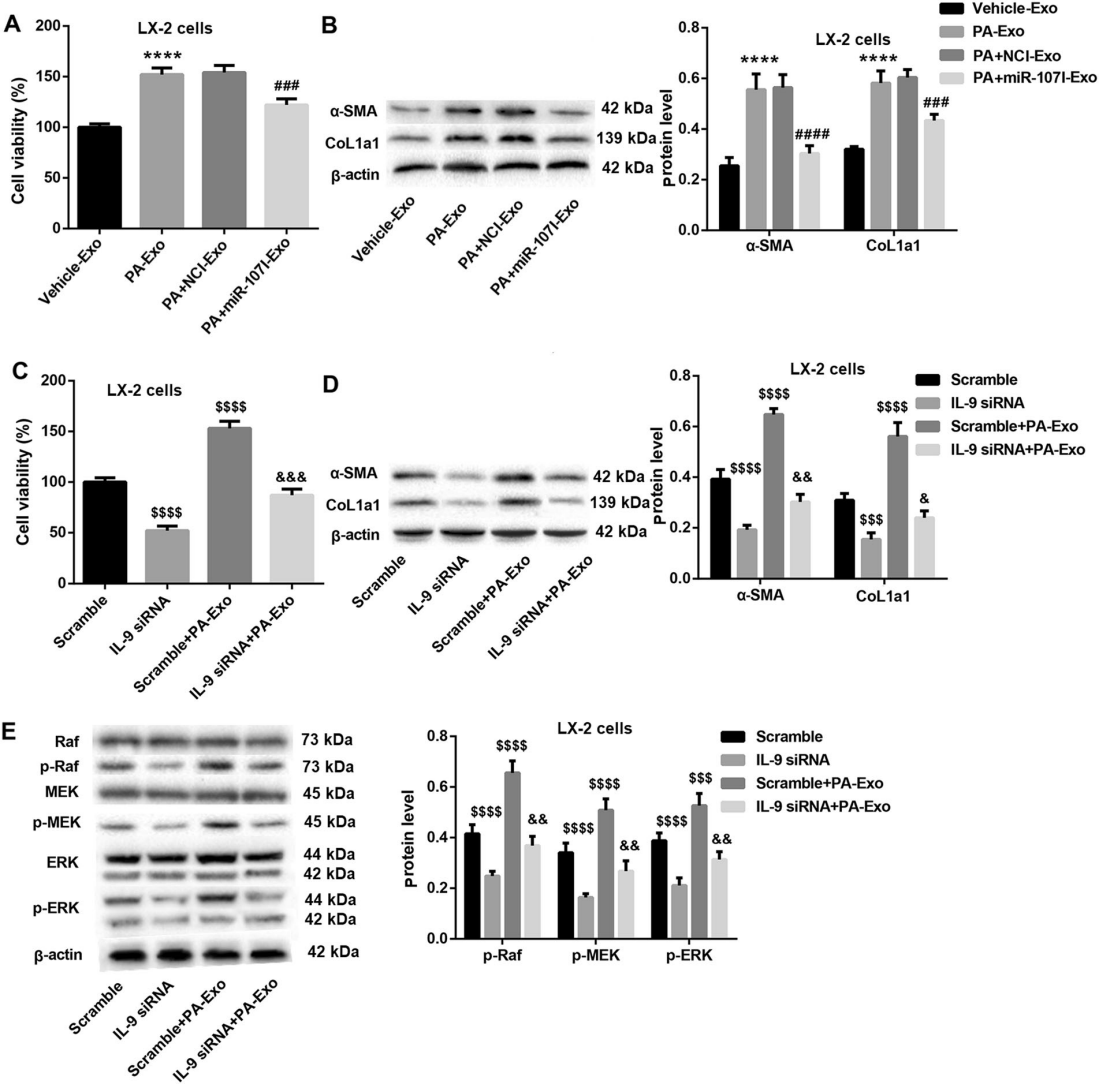
PA-Exo promotes LX-2 cell activation through miR-107-mediated upregulation of IL-9 expression in CD4^**+**^ T cells. CD4^+^ T cells were cocultured with hepatocytes-derived exosomes (Vehicle-Exo, PA-Exo, PA + NCI-Exo, and PA + miR-107I-Exo). Then the culture media were collected and added into culture media of LX-2 cells. CCK-8 and western blot were carried out to evaluate cell viability (**A**) and the protein levels of α-SMA and CoL1a1 (**B**) in LX-2 cells. CD4^+^ T cells were transfected with IL-9 siRNA or scramble control, followed by PA-Exo treatment. CCK-8 and western blot were carried out to evaluate cell viability (**C**), the protein levels of α-SMA and CoL1a1 (**D**), and the protein levels of Raf, p-Raf, MEK, p-MEK, ERK, and p-ERK (**E**) in LX-2 cells. ^****^P < 0.0001 vs. Vehicle-Exo; ^###^*P* < 0.001, ^####^*P* < 0.0001 vs. PA + NCI-Exo; ^$$$^*P* < 0.001, ^$$$$^*P* < 0.0001 vs. Scramble; ^&^*P* < 0.05, ^&&^*P* < 0.01, ^&&&^*P* < 0.001 vs. IL-9 siRNA. Data were expressed as the mean ± standard deviation (*n* = 3).

To determine whether PA-Exo also regulates LX-2 cell activation by transferring miR-107 to CD4^+^ T cells, we cocultured CD4^+^ T cells with Vehicle-Exo, PA-Exo, PA + NCI-Exo, and PA + miR-107I-Exo, after which the co-culture media was collected and then added into the culture of LX-2 cells. Data revealed that, compared with the Vehicle-Exo group, the cell viability (Fig. 7A) and protein levels of α-SMA and CoL1a1 (Fig. 7B) in LX-2 cells in the PA-Exo group were significantly elevated, indicating that co-culture media from PA-Exo and CD4^+^ T cells can induce LX-2 cell activation. Of note, PA + miR-107I-Exo group displayed a significant reduction in LX-2 cell activation (Fig. 7A, B). These data indicated that PA-Exo might induce LX-2 cell activation by transferring miR-107 to CD4^+^ T cells.

Based on the data in Fig. 6, therefore, we guessed whether PA-Exo induced LX-2 cell activation by regulating IL-9 expression in CD4^+^ T cells. Data revealed that IL-9 silencing in CD4^+^ T cells significantly inhibited cell proliferation (Fig. 7C) and decreased protein levels of α-SMA and CoL1a1 (Fig. 7D) in LX-2 cells, indicating that IL-9 silencing in CD4^+^ T cells inhibited LX-2 cell activation. Considering that IL-9 promoted liver fibrosis via Raf/MEK/ERK signaling pathway [18], Raf/MEK/ERK pathway was also examined. As expected, the significant decreased phosphorylation levels of Raf, MEK, and ERK were observed in CD4^+^ T cells with IL-9 silencing (Fig. 7E). More importantly, IL-9 knockdown in CD4^+^ T cells partially reversed the obviously enhanced activation of LX-2 cells and Raf/MEK/ERK signaling pathway mediated by PA-Exo in LX-2 cells. These data indicated that PA-Exo promoted LX-2 cell activation, at least in part, through transferring miR-107 to upregulate IL-9 expression and activate Raf/MEK/ERK signaling in CD4^+^ T cells.

### IL-9 promotes LX-2 cell activation through activating Raf/MEK/ERK pathway

**Fig. 8:**
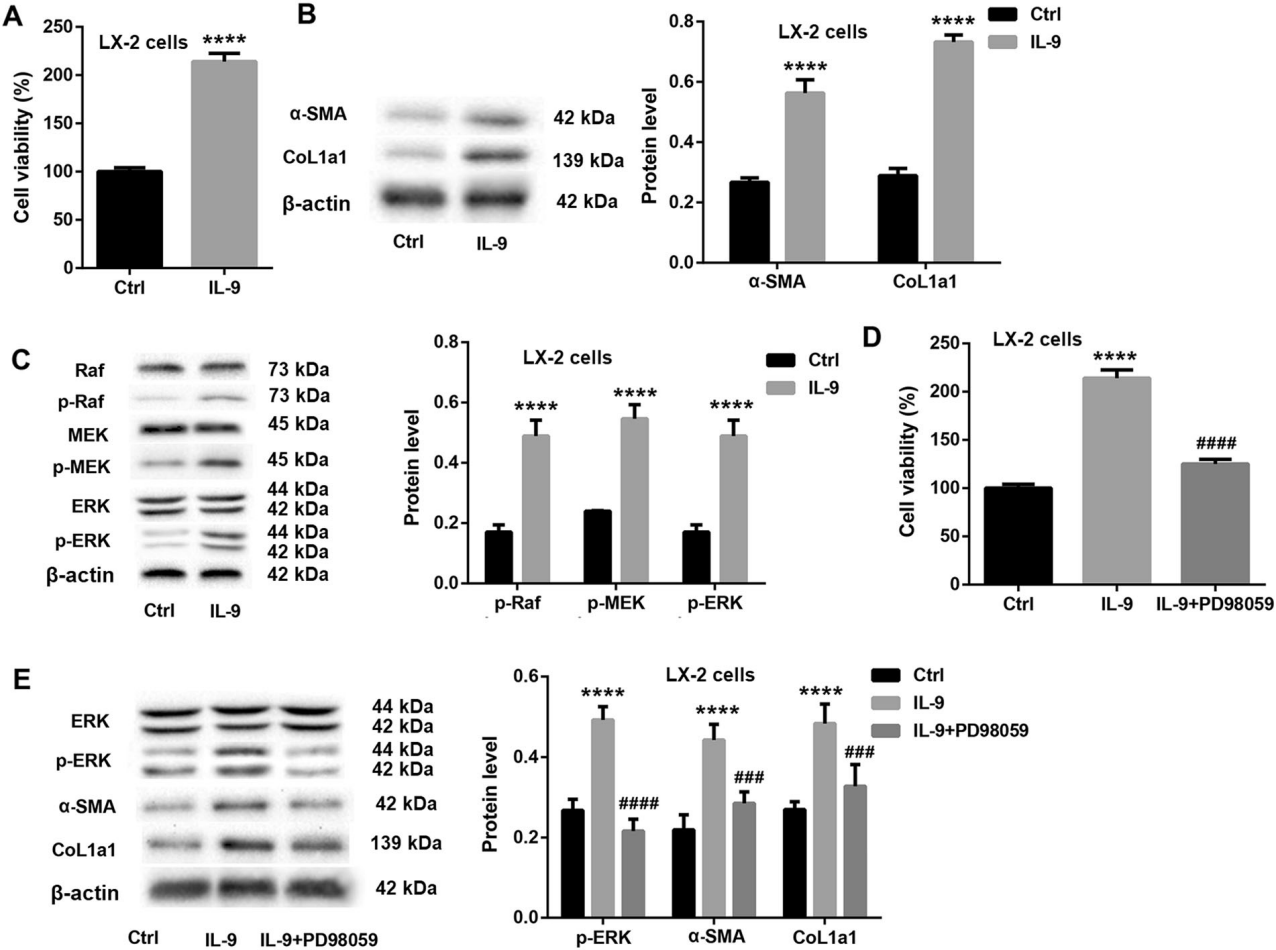
IL-9 promotes LX-2 cell activation through activating Raf/MEK/ERK pathway. LX-2 cells were transfected with IL-9 overexpression vector or control empty vector. CCK-8 and western blot were carried out to evaluate cell viability (**A**), the protein levels of α-SMA and CoL1a1 (**B**), and the protein levels of Raf, p-Raf, MEK, p-MEK, ERK, and p-ERK (**C**) in LX-2 cells. LX-2 cells were transfected with IL-9 overexpression vector or control empty vector, followed by PD98059 treatment. CCK-8 and western blot were carried out to evaluate cell viability (**D**), the protein levels of ERK, p-ERK, α-SMA, and CoL1a1 (**E**) in LX-2 cells. ^****^P < 0.0001 vs. Ctrl; ^###^*P* < 0.001, ^####^*P* < 0.0001 vs. IL-9. Data were expressed as the mean ± standard deviation (*n* = 3).

Furthermore, we also explored whether IL-9 promoted LX-2 cell activation through activating Raf/MEK/ERK pathway. The results showed that IL-9 overexpression significantly facilitated cell proliferation (Fig. 8A), elevated the protein levels of α-SMA and CoL1a1 (Fig. 8B), as well as the phosphorylation levels of Raf, MEK, and ERK (Fig. 8C) in LX-2 cells, indicating that IL-9 induced LX-2 cell activation and Raf/MEK/ERK signaling. However, PD98059, an inhibitor of Raf/MEK/ERK signaling, effectively abrogated the IL-9 overexpression-mediated increased cell proliferation (Fig. 8D) and protein levels of α-SMA and CoL1a1 (Fig. 8E), indicating that IL-9 promoted LX-2 cell activation through activating Raf/MEK/ERK pathway.

## Discussion

HSCs activation is a prominent driver of liver fibrogenesis in various chronic liver disorders including NAFLD [15]. Currently, the trigger for HSCs activation in NAFLD remains not entirely clear. Certain lipids, such as saturated FFAs, have toxic effects on hepatocytes. PA, a kind of saturated FFA, is often used to simulate the pathogenesis of NAFLD and induce liver cell damage for in vitro experiments in many studies [19, 20]. Thus, here, we treated the cells with PA to induce liver cell damage to mimic the pathogenesis of NAFLD. It has been reported that exosomes secreted by PA-treated hepatocytes elevated the expression of fibrotic genes in HSCs [10, 21]. Similarly, in our study, exosomes derived from PA-treated hepatocytes markedly induced cell proliferation and upregulated profibrogenic genes (α-SMA and CoL1a1) expression in LX-2 cells, which are markers of HSCs activation.

The important issue that needs to be addressed is how exosomes from PA-treated hepatocytes induce HSCs activation? Convincing evidence indicates that exosomes exihibit important roles between hepatocytes and HSCs [10]. For example, hepatocytes secrete exosomes during liver injury, which mediate the activation of Toll-like receptor 3 (TLR3) in HSCs and thus exacerbate liver fibrosis by enhancing interleukin-17A (IL-17) production by γδ T cells [22]. This supports the notion that hepatocytes can modulate HSCs phenotypical switch and liver fibrosis by delivering specific molecules (particularly miRNAs) via exosomes. A previous study showed that extracellular vesicles released from PA-treated hepatocytes were efficiently internalized by HSCs, and resulting in their activation by miR-128-3p delivery [21]. However, intercellular signal transduction between exosomes and miRNA in NAFLD still remain incompletely understood.

MiR-107 is associated with NASH progression, fibrosis, and hepatocarcinoma [11, 23]. Our results confirmed that miR-107 was abundant in PA-treated hepatocytes derived exosomes, which was consistent with the results of a previous study using microarray analysis [10]. Furthermore, miR-107 expression was also significantly upregulated in LX-2 cells after PA-Exo treatment, compared with Vehicle-Exo group. Notably, the transfection with miR-107I into hepatocytes effectively abrogated the PA-Exo-induced LX-2 cell activation. These data collectively indicated that exosomes from PA-treated hepatocytes induced LX-2 cell activation by transferring miR-107 to LX-2 cells.

Wnt signaling has been shown to facilitate liver fibrosis by enhancing HSCs activation [13]. Our luciferase reporter assay confirmed that miR-107 targeted DKK1, an antagonist of Wnt pathway [12]. Further results in this study demonstrated that PA-treated hepatocytes-derived exosomes shuttled miR-107 into LX-2 cells, resulting in the inhibition of DKK1 and consequent activation of Wnt signaling, and thus activating LX-2 cells.

HSCs activation can also be regulated by immune cells. T lymphocytes play a key role in modulating HSCs activation and hepatic fibrosis by secreting immunomodulatory factors such as IL-17 and IL-22 [24, 25]. Th9 cells are a new subset of Th cells and characterized by high levels of the signature cytokine IL-9 [26]. In a CCl4-induced mouse model of hepatic fibrosis, compared with the controls, significantly elevated Th9 cells in spleen, and high expression of IL-9 in plasma and liver were all observed [16]. Furthermore, IL-9 neutralization in mice with hepatic fibrosis significantly reduced Th9 cells in spleen, and suppressed IL-9 expression in plasma and liver, attenuated HSCs activation, and ameliorated hepatic fibrosis [16], suggesting that Th9/IL-9 axis facilitated HSCs activation in hepatic fibrosis. Our data also revealed that PA-Exo induced Th9 cell differentiation and IL-9 expression in CD4^+^ T cells to activate LX-2 cells by transferring miR-107 to CD4^+^ T cells. In addition, the luciferase reporter assay confirmed that Foxp1 was also a target of miR-107 in CD4^+^ T cells. Furthermore, our investigation on the mechanism by which PA-Exo transferred miR-107 to induce LX-2 cell activation also showed that, exosomes released from PA-treated hepatocytes delivered miR-107 to CD4^+^ T cells, where miR-107 targeted Foxp1, which inhibited Th9 cell differentiation and IL-9 expression via binding to the IL-9 promoter in CD4^+^ T cells, and the increased IL-9 expression promoted LX-2 cell activation by activating the Raf/MEK/ERK signaling pathway in CD4^+^ T cells.

In summary, our findings demonstrated that PA-treated hepatocytes-derived exosomes promoted LX-2 cell activation by transferring miR-107. The underlying mechanisms were associated with the direct inhibition of Dickkopf-1 (DKK1) by miR-107 in LX-2 cells, as well as the targeted regulation of Forkhead box protein P1 (Foxp1) by miR-107 in CD4^+^ T cells, which was illustrated in Supplementary 2. Our findings emphasize the important role of exosomes in the progression of NAFLD and identify a potential molecular target for anti-fibrotic therapeutic interventions.

## Materials and Methods

### Human hepatocytes isolation and palmitic acid treatment

The human normal hepatocytes were obtained from the surgically removed normal liver tissue by collagenase, and informed consent was obtained from all the subjects. To induce lipid accumulation, hepatocytes were treated with 50 μg/mL palmitic acid (PA; 0.4 mM; Sigma Aldrich) for 16 h. All the experimental protocols were approved by the Ethics Committee of The Second Affiliated Hospital of Nanchang University.

### Isolation and identification of hepatocytes-derived exosomes

Exosomes were isolated from the supernatant of hepatocytes by ultrahigh velocity centrifugation. Firstly, culture media samples were centrifuged at 2000×*g* for 10 min to discard dead cells. The cell supernatant was then centrifuged at 10,000×*g* for 30 min to remove cell debris. Afterward, the supernatant was subject to additional centrifugation at 100,000×*g* for 70 min. The precipitated exosome pellets were resuspended in PBS for indentification using transmission electron microscopy (TEM), nanoparticle tracking analysis, and western blot as described elsewhere [10].

### Grouping of exosomes

The hepatocytes-derived exosomes were divided into vehicle-Exo, PA-Exo, PA + NCI -Exo, and PA + miR-107I-Exo group. Exosomes isolated from the culture supernatant of hepatocytes which were pretreated with vehicle (1% endotoxin-free, low-FFA bovine serum albumin) or 0.4 mM PA (dissolved in 95% ethanol) for 16 h were namely vehicle-Exo and PA-Exo, respectively. The hepatocytes were transfected with miR-107 inhibitor (miR-107I) or inhibitor negative control (NCI), and followed by 0.4 mM PA treatment for 16 h. Exosomes were isolated from the culture supernatant of these hepatocytes, namely, PA + NCI -Exo, and PA + miR-107I-Exo, respectively.

### Co-culture of hepatocytes-derived exosomes and LX-2 cells

LX-2 cells provided by BeNa Culture Collection (Beijing, China) were cultured in DMEM (Gibco) supplemented with 10% FBS (Gibco) with 5% CO_2_ at 37 °C. LX-2 cells were cocultured with exosomes for 16 h.

### Quantitative real-time polymerase chain reaction (qRT-PCR) analysis

Total RNA isolated from cells was reverse transcribed into cDNAs using a High Capacity cDNA Reverse Transcription Kit (Applied Biosystems, Foster City, CA, USA). The cDNA template was synthesized through qRT-PCR using the SYBR green dye (Applied Biosystems). The expression of genes were calculated by the 2^-ΔΔCt^ method. The miR‐107 expression was normalized to U6, and expression levels of DKK1, interleukin (IL)−9, and Foxp1 were normalized to GAPDH. The sequences of primers were listed in Supplementary Table S1.

### Cell viability assay

Cell viability was determined using the Cell Counting Kit-8 (CCK-8; Dojindo, Kumamoto, Japan), and the optical density (OD) at 450 nm was measured by a microplate reader (BioTek Instruments, Inc., Winooski, VT, USA).

### Western blot

Briefly, equivalent aliquots of protein (40 μg) were loaded per lane and separated using 10% SDS-PAGE gel electrophoresis, then transferred to PVDF membranes. After being blocked in 5% nonfat milk for 2 h, the membranes were incubated with primary antibodies against α-SMA (#19245), CoL1A1 (#72026), DKK1(#482367), β-catenin (#8480), c-myc (#18583), cyclinD1 (#55506), Raf (#9422), phospho (p)-Raf (#9421), MEK (#12671), p-MEK (#98195), ERK (#4695), p-ERK (#4370) (all purchased form Cell Signaling Technology, Beverly, MA, 1:1000) overnight at 4 °C. The membranes were incubated with the horseradish peroxidase (HRP)-conjugated secondary antibodies (1:2000; Santa Cruz Biotechnology Inc.) at room temperature for 2 h. Image-Pro Plus 6.0 software was used to analyze the immunoreactive bands. β-actin was used as the loading control.

### Luciferase reporter assay

Dual luciferase reporter assays were performed to verify the direct interactions between miR-107 and DKK1 as well as miR-107 and Foxp1. Briefly, DKK1 3’UTR or Foxp1 3′UTR containing the predicted wild-type (WT) or mutated (Mut) binding sites of miR-107 were cloned into pGL3 vector. Then, the reporter vector constructs were co-transfected with mimic NC or miR-107 mimic into HEK293T cells using Lipofectamine^TM^ 2000. Twenty-four hours after transfection, the luciferase activity was analyzed using a Dual-Luciferase Reporter Assay Kit (Promega Corporation, Madison, WI, USA).

### Cell transfection

To overexpress DKK1 and IL-9, the full-length DKK1 and IL-9 cDNA fragments were cloned into pcDNA 3.1 (Invitrogen, USA), named pcDNA3.1-DKK1 and pcDNA3.1-IL-9, respectively. SiRNA targeting IL-9 (IL-9 siRNA), miR-107 mimic, miR-107 inhibitor, and their corresponding controls were purchased from GenePharma (Shanghai, China). Cells were transfected using Lipofectamine 3000 according to the manufacturer’s protocols (Invitrogen, Carlsbad, CA).

### Isolation of naive CD4^+^ T cells

Isolation of naive CD4^+^ T cells was performed as previously described [27]. Briefly, peripheral blood mononuclear cells (PBMCs) were purified from freshly collected, heparinized peripheral blood of healthy donors by FicollHypaque density gradient centrifugation. Naive CD4^+^ T cells were isolated from PBMCs by negative selection for CD45RA expression using the naive CD4^+^ T cell isolation kit II (Miltenyi Biotec). CD4^+^ T cells were cocultured with exosomes for 16 h.

### In vitro Th9 cell differentiation

Naive CD4^+^ T cells were isolated and cocultured with vehicle-Exo, PA-Exo, NCI-Exo, and miR-107I-Exo, respectively. Th9 differentiation was induced with human IL-4 (10 ng/mL, R&D Systems), human TGF-β (1 ng/mL, R&D Systems), and anti-IFN-γ monoclonal antibodies (10 μg/mL, eBioscience) for 3 days of culture.

### Detection of Th9 proportion by flow cytometry

The proportion of Th9 cells (IL-9^+^ CD4^+^ T cells) to CD4^+^ T cells was detected using flow cytometry. Polarized Th cells were stimulated with phorbol ester (PMA, 50 ng/mL; Sigma-Aldrich), ionomycin (500 ng/mL; Sigma-Aldrich) and blocked by brefeldin A (BFA, BioLegend) for 4 h. Then, cells were stained with FITC-conjugated anti-CD4 antibody at 4 °C, fixed, and permeabilized with the BD Cell Fixation/Permeabilization Kit, followed by incubation with PE-conjugated anti-IL-9 antibody at 4 °C in the dark. Cells were analyzed with a BD FACSCalibur flow cytometer (BD Bioscience, San Jose, CA, USA) equipped with FlowJo software (TreeStar).

### Chromatin immunoprecipitation (ChIP) assay

ChIP assay was performed using SimpleChIP Enzymatic Chromatin IP Kit (Cell Signaling Technology) according to the manufacturer’s instructions. Briefly, CD4^+^ T cells were cross-linked with 1% formaldehyde for 10 min at room temperature, harvested, and resuspended in lysis buffer. Following sonication and centrifugation, sheared chromatin was incubated overnight at 4 °C with anti-Foxp1 (Abcam, USA). Serum IgG served as a negative control.

### Statistical analysis

GraphPad Prism 7.0 was adopted to perform statistical analyses. The differences between groups were analyzed using the unpaired Student’s *t*-test or one-way analysis of variance (ANOVA). * for a significant difference less than *P* < 0.05, ** for a significant difference less than *P* < 0.01, *** for a significant difference less than *P* < 0.001; **** for a significant difference less than *P* < 0.0001.

## Supplementary information

Supplementary Table S1

Supplementary 1

Supplementary 2

## Data Availability

The data that support the findings of this study are available from the corresponding author upon reasonable request.
