# Supplementary material for: Exosomes secreted by palmitic acid-treated hepatocytes promote LX-2 cell activation by transferring miRNA-107
Source: Cell Death Discov. 2021 Jul 7;7:174. doi: 10.1038/s41420-021-00536-7 (PMC8263701; doi:10.1038/s41420-021-00536-7)
Supplement: Supplementary file 1 — Supplementary Table S1 [file 41420_2021_536_MOESM1_ESM.doc]

**Supplementary Table S1. List of primer sequence that used in qRT-PCR analysis**

| **Primer name** | **Forward (5’-3’)** | **Reverse (5’-3’)** |
| --- | --- | --- |
| miR-107 | AAAGAATTCCTGTTTCACTCGCCAAGC | AAAGGATCCAGCGAGTGAGGAGGGAGA |
| U6 | GCTTCGGCAGCACATATACTAAAAT | CGCTTCACGAATTTGCGTGTCAT |
| DKK1 | TAGAGTCTAGAATGCAAGGATCTC | CAAAAACTATCACAGCCTAAAGGG |
| IL-9 | CTCTGTTTGGGCATTCCCTCT | GGGTATCTTGTTTGCATGGTGG |
| Foxp1 | CAGCGAAACCACGAAAAGAAG | GGTCCACCTCTGTTAGTGATA |
| GAPDH | GGAGCGAGATCCCTCCAAAAT | GGCTGTTGTCATACTTCTCATGG |
