# Supplementary figures and images for: Exosomes secreted by palmitic acid-treated hepatocytes promote LX-2 cell activation by transferring miRNA-107
Source: Cell Death Discov. 2021 Jul 7;7:174. doi: 10.1038/s41420-021-00536-7 (PMC8263701; doi:10.1038/s41420-021-00536-7)

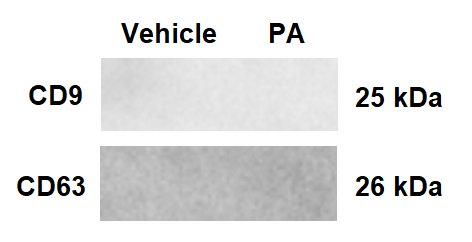

Supplement: Supplementary file 2 — Supplementary 1 [file 41420_2021_536_MOESM2_ESM.tif]

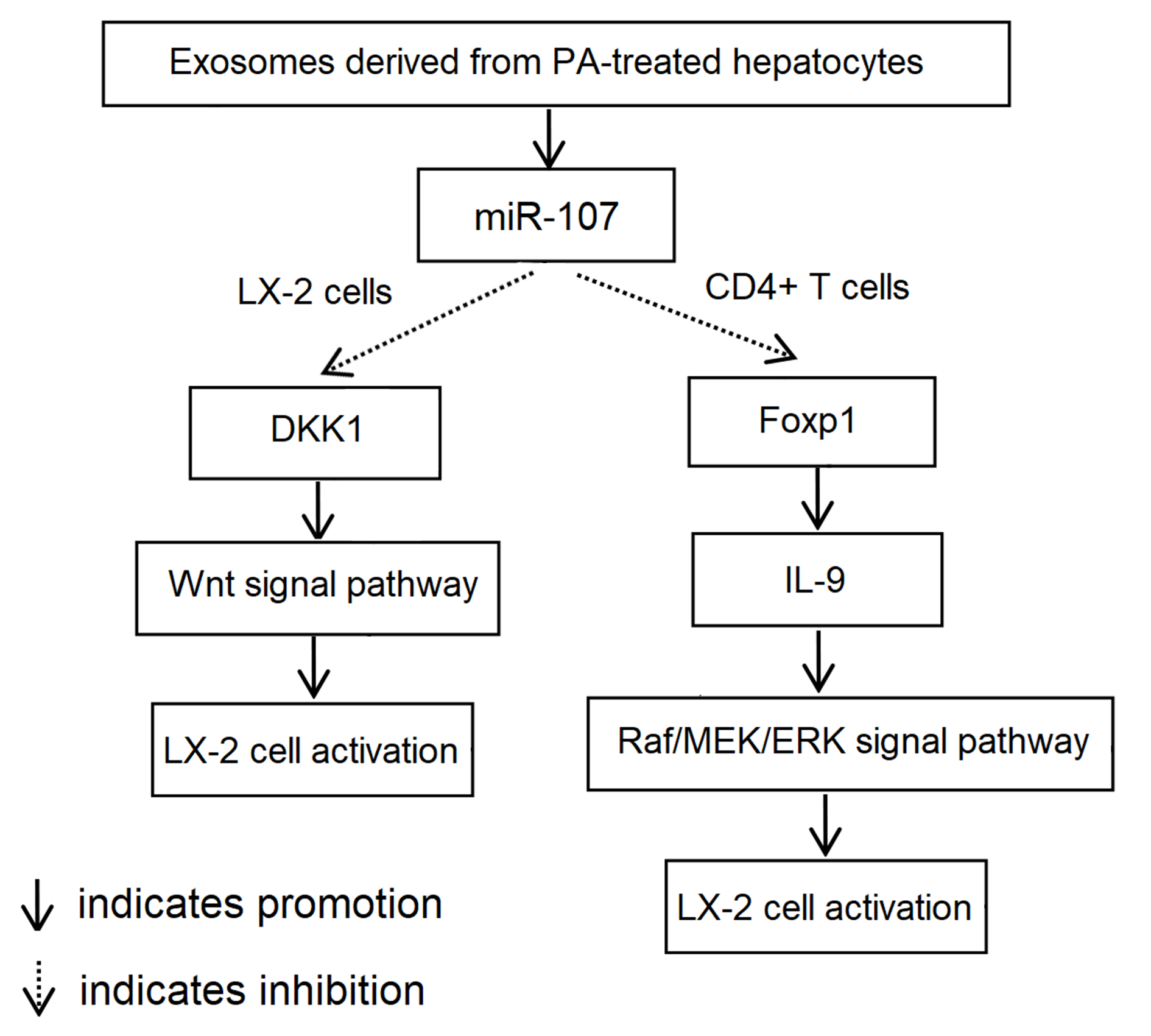

Supplement: Supplementary file 3 — Supplementary 2 [file 41420_2021_536_MOESM3_ESM.tif]
